# Perceptions and Impacts of COVID-19 on Family Caregivers

**DOI:** 10.1093/geroni/igab046.310

**Published:** 2021-12-17

**Authors:** Alexa Balmuth

**Affiliations:** MIT AgeLab, Cambridge, Massachusetts, United States

## Abstract

For many family caregivers, COVID-19 has presented new obstacles to providing elder-care while balancing additional responsibilities such as work or childcare. Three survey waves explored impacts over the course of the pandemic. Family caregivers demonstrated resilience, taking a variety of measures to care for and protect family; caregivers were also more confident in their ability to protect loved ones age 60+ from contracting COVID-19 than non-caregivers. However, COVID-19’s toll on caregivers was evident. Caregivers reported higher personal burnout than non-caregivers, and across all three survey waves, consistently reported greater degrees of worry in regards to COVID-19 in general, as well as its impacts on domains including the health and wellbeing of themselves and family members, and near and far term finances. This presentation will highlight how caregivers’ perceptions and impacts of COVID-19 have evolved over the course of the pandemic, and implications of these findings for life tomorrow.

